# Soluble urokinase-type plasminogen activator receptor strongly predicts global mortality in acute heart failure patients: insight from the STADE-HF registry

**DOI:** 10.2144/fsoa-2020-0197

**Published:** 2021-03-29

**Authors:** Fabien Huet, Anne-Marie Dupuy, Claire Duflos, Cintia Azara Reis, Nils Kuster, Sylvain Aguilhon, Jean-Paul Cristol, François Roubille

**Affiliations:** 1 Cardiology Department, University Hospital of Montpellier, Montpellier, France; 2 PhyMedExp, University of Montpellier, INSERM U1046, CNRS UMR, 9214, Montpellier, 34090, France; 3 Department of Biochemistry, Centre Ressources Biologiques de Montpellier, University Hospital of Montpellier, Montpellier, 34090, France; 4 CEPEL, Univ Montpellier, CNRS, Montpellier, 34090, France; 5 Medico-Economic Research Unit, CHU Montpellier, University of Montpellier, Montpellier, 34090, France

**Keywords:** acute heart failure, biomarkers, prognosis, suPAR

## Abstract

**Background:**

Whether soluble urokinase-type plasminogen activator receptor (suPAR) could be a valuable prognostic indicator remains uncertain.

**Materials & methods:**

Patients from STADE-HF (Soluble Suppression of Tumorigenesis-2 as a Help for Management of Diagnosis, Evaluation and Management of Heart Failure) were included for analysis.

**Results:**

95 patients were included. The suPAR level of expression was significantly higher in the group of patients who died at one month (7.90 ± 4.35 ng/ml vs 11.94 ± 6.86 ng/ml; p < 0.05) or 1 year (7.28 ± 4.27 ng/ml vs 11.81 ± 4.88 ng/ml; p < 0.01), but there was no significant difference according to the readmission.

**Conclusion:**

High suPAR levels during hospitalization for acute heart failure were highly predictive for the risk of mortality, but not the risk of readmission.

## Lay abstract

Finding the best biomarker to evaluate prognosis in heart failure is challenging. Our objective is to evaluate the soluble urokinase-type plasminogen activator receptor prognosis performance in acute heart failure (AHF) patients on global mortality, risk of readmission after an acute heart failure, using the STADE-HF cohort of patients. The soluble urokinase-type plasminogen activator receptor level of expression was significantly higher in the group of patients who died during follow-up, but there was no significant difference according to the readmission for heart failure. This could help to stratify patients.

Heart failure (HF) is a leading cause of hospitalization, morbidity, mortality and represents a tremendous burden to healthcare systems [1]. Although long-term treatments have proved beneficial in reducing hospitalization and mortality, acute HF exacerbations leading to hospital (re)admissions remain frequent and the main determinant of costs [1]. Reducing readmission rate appears then crucial. Identifying patients at risk of readmission or poor outcome is challenging [2]. Consistently, guidelines recommend to evaluate myocardial fibrosis biomarkers (including soluble suppression of tumorigenesis-2 [sST2]) to stratify the cardiovascular risk of patients admitted for acute HF decompensation [3]. sST2 is a member of the IL-1 receptor family, secreted in response to biomechanical stress and inflammation [4–7]. sST2 was identified as a new prognostic biomarker in HF, independent from age, kidney function, anemia or BMI [6,8]. Recently, our team demonstrated that another biomarker GDF-15 added substantial information on patients’ prognosis [9]. Urokinase-type plasminogen activator receptor (UPAR) is a glycoprotein involved in tissue reorganization events and regulation of plasminogen [10,11]. Under inflammatory stimulation the protein detaches from cell surface and transform into its soluble form (soluble urokinase-type plasminogen activator receptor [suPAR]), increasing its serum level. High suPAR serum levels are found in case of HF, paving the way for its use as a prognosis biomarker in AHF. Whether suPAR levels decrease with effective therapy and whether suPAR can serve as a valuable prognostic indicator remains uncertain. Our objective is to evaluate the suPAR prognosis performance in AHF patients on global mortality, risk of readmission after acute HF.

## Materials & methods

STADE-HF (sST2 as a help for management of diagnosis, evaluation and management of HF) was a single-blinded prospective randomized controlled trial conducted at University Hospital of Montpellier (Internal Medicine and Cardiology Departments) [12]. It included patients hospitalized for acute HF, and the sST2 was measured at J0 and J4. In the intervention group (ST2 group), the treatment was optimized following the value of the sST2; in the control group (BNP group), the cardiologists was blinded to the sST2 and optimized the treatment following usual practice. The design paper was published elsewhere [13] and referenced in clinical trial (NCT02963272).

### Population selection

All patients admitted to our institution between January 2017 and August 2018 for acute HF, with a N-terminal pro B-type natriuretic peptide (NTproBNP) ≥450 pg/ml (BNP ≥400 pg/ml) at any moment of the hospitalization, or necessity to increase or introduce a diuretic treatment (orally or intravenously) were included in this study. Acute HF decompensation followed the ESC 2016 definition and was independent of left ventricular ejection fraction (LVEF) value. Exclusion criteria were patients <18 years of age, hemodynamic instability, life expectancy <7 days, participation in another study, pregnancy or nursing, patients under justice safeguard, those under protection or under curatorship, and those from whom we were unable to get clear information. All participants signed a written consent form and were aware of their right to withdraw from the study at any time.

### Methods

Patients from STADE-HF study had their data retrospectively assessed with the intention of comparing severity and outcomes in patients with HF, according to the serum level of suPAR. suPAR level was measured at patient admission, regardless of study group. Initially, we included 123 participants in the STADE-HF study [12]. Inside this population we could analyse the suPAR level in 47 patients from the control group and 50 patients in the sST2 group. Two patients withdrew consent during follow-up (one in the low sST2 group and one in the control group). The randomization was centralized, stratified on sex, age and glomerular filtration rate (below 30, between 30 and 60 and above 60 ml/min), with varying block sizes unknown to the investigators, and performed using a web-based interface.

### End points

The main clinical end point was the readmission rate for any cause at 1 month and 1 year. Secondary clinical end points were the risk of all-cause death at 1 month and 1 year, the rehospitalization rate for acute HF decompensation at 1 month and 1 year, the duration of initial hospitalization, a tolerance criteria based on the evaluation of kidney function at 1 month after discharge. Endopoint data were collected during a standardized and systematic 1-month follow-up consultation, and by patient files examination, of by phone call for the 1-year events.

### Blood sample tests

At inclusion of patients, routine parameters such as urea, electrolytes, creatinine, N terminal pro brain natriuretic peptide (NT-proBNP), hs-cTnT and C-reactive protein (CRP), were performed on Cobas 8000 (Roche Diagnostic, Meylan, France) using e701 and e602 modules. In addition, venous blood was collected in dry and ethylenediaminetetraacetic acid tubes, immediately centrifuged and frozen (-80°C) on several aliquots until tested. sST2 plasma concentrations were measured with ASPECT-PLUS ST2 test, a rapid quantitative lateral flow immunoassay with the ASPECT reader (Critical Diagnostics, CA, USA [distributed in France by Eurobio society]) using an ethylenediaminetetraacetic acid plasma aliquot never thawed. In june 2019, the determination of suPAR levels (from plasma aliquot never thawed) was performed with suPARnostic©TurbiLatex reagents provided by ViroGates society (Birkerød, Denmark) and adapted on Cobas 8000 analyzer (module c502) using a turbidimetric method.

### Statistical analysis

All variables were described in the whole sample and in subgroups, using mean and standard deviation for continuous values, and number and percentages for the categorical variables. Continuous variables were compared between groups using Student’s t test when possible, or using Wilcoxon–Mann–Whitney’s test. Qualitative variables were compared using Chi-square’s test when possible, or using Fisher’s exact test. The prognosis performance of the suPAR was assessed by the area under the receiver operating characteristic (ROC) curve (area under the curve), and by its 95% CI. The added prognosis performance of the suPAR, relatively to known prognostic factors, was assessed using multivariate logistic regression. The first model included clinical factors (age, sex, LVEF) in addition to suPAR, and the second model included biological factors (CRP, NT-proBNP and ST2) in addition to suPAR. If the linearity condition was not met, variables were dichotomized according to their median in our analysis sample. No variables selection was done in multivariate analysis.

## Results

### Study population: 95 patients from the STADE HF randomized trial

**Figure 1.**
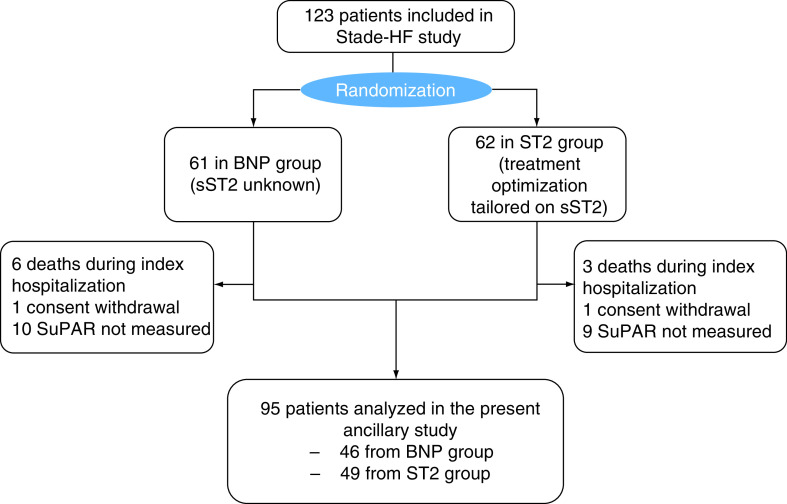
Study flow chart. The present study is an ancillary study of a randomized trial: NCT02963272. Patients from STADE-HF study had their data retrospectively assessed. suPAR level was measured at patient admission, regardless of study group. Initially, we included 123 participants in the STADE-HF study. Inside this population we could analyze the suPAR level in 47 patients from the control group and 50 patients in the sST2 group. Two patients withdrew consent during follow-up (one in the low sST2 group and one in the control group). sST2: Soluble suppression of tumorigenesis-2; suPAR: Soluble urokinase-type plasminogen activator receptor.

Ninety-five patients were included in the statistical analysis. The median age was 77.00 (64.00; 84.00) years, with 56 (58.9%) of men. Main etiologies of HF were rhythmic, ischemic, valvular and hypertensive cardiomyopathies. The median LVEF was 43.00% (30.00; 50.00) and the New York Heart Association at inclusion was of stage 3 in 93 patients (56.1%). The mean level of suPAR expression in the global population was 8.28 ± 4.76 ng/ml. Patients baseline characteristics are summarized in Table 1. Flow chart is in Figure 1.

**Table 1.** Patients baseline characteristics.

Variable	Total population (n = 95)
Male gender, n (%)	56 (58.9)
Age (years), median (95% CI)	77.00 (64.00; 84.00)
BMI, median (95% CI)	27.41 (24.54; 30.94)
Smokers, n (%)	13 (13.68)
Diabetes mellitus, n (%)	36 (37.89)
Dyslipidemia, n (%)	26 (27.37)
NYHA scale, n (%)	
I	1 (1.05)
II	8 (8.42)
III	58 (61.05)
IV	28 (29.47)
Etiology of cardiopathy	
Ischemic, n (%)	34 (35.79)
Hypertensive, n (%)	11 (11.58)
Valvular, n (%)	33 (34.74)
Arrhythmic, n (%)	50 (52.63)
LVEF, median (95% CI)	43.00 (30.00; 50.00)
Pulmonary hypertension, n (%)	48 (51.06)
Creatinine clearance, median (95% CI)	54.00 (34.00; 69.00)
NT-proBNP, median (95% CI)	4091.00 (2310.00; 7788.00)
sST2 level, median (95% CI)	112.00 (51.40; 250.00)
sUPAR level, median (95% CI)	7.67 (4.30; 10.72)

LVEF: Left ventricular ejection fraction; NT-proBNP: N terminal pro brain natriuretic peptide; NYHA: New York Heart Association; sST2: Soluble suppression of tumorigenesis-2; sUPAR: Soluble urokinase-type plasminogen activator receptor.

### Primary criterion outcomes

At 1 month, 10 patients (10.35%) died, 17 patients (20.24%) were hospitalized, including eight patients (9.52%) readmitted for HF. At 1 year, 22 patients (23.16%) died and 25 patients (29.07%) were hospitalized, including 14 patients (16.28%) readmitted for HF.

### Univariate analysis for suPAR level & outcomes at one month

At 1 month, the suPAR level of expression was significantly higher in the group of patients who died (7.90 ± 4.35 ng/ml vs 11.94 ± 6.86 ng/ml; p < 0.05), but there was no significant difference according to the readmission on the first month (8.09 ± 4.58 ng/ml vs 6.97 ± 3.18; p = 0.61); similarly no difference according readmission for heart failure within the first month (8.01 ± 4.41 ng/ml vs 6.53 ± 3.68; p = 0.37, respectively).

### Univariate analysis for suUPAR level & outcomes at one year

At 1 year, the suPAR level of expression was significantly higher in the group of patients who died (7.28 ± 4.27 ng/ml vs 11.81 ± 4.88 ng/ml; p < 0.01), but there was no significant difference according if the patients were readmitted during the first year (7.90 ± 4.71 ng/ml) or not (7.94 ± 3.29; p = 0.46). There was no significant difference according if the patients were readmitted for heart failure during the first year (7.83 ± 4.44 ng/ml) or not (8.34 ± 3.81; p = [0.46]). The median of the suPAR was 7.54 ng/ml. We separated the patients into two subgroups: high level (49 patients with SUPAR >7.54 ng/ml) or low (46 patients with suPAR <7.54 ng/ml). In the group of patients with a high level of suPAR expression, the rate of death of any cause was higher at 1 month (16.33 vs 4.35%; p = 0.09) and at 1 year (38.78 vs 6.52%; p = 0.01).

### ROC curves for high suPAR level predict the risk of death.

**Figure 2.**
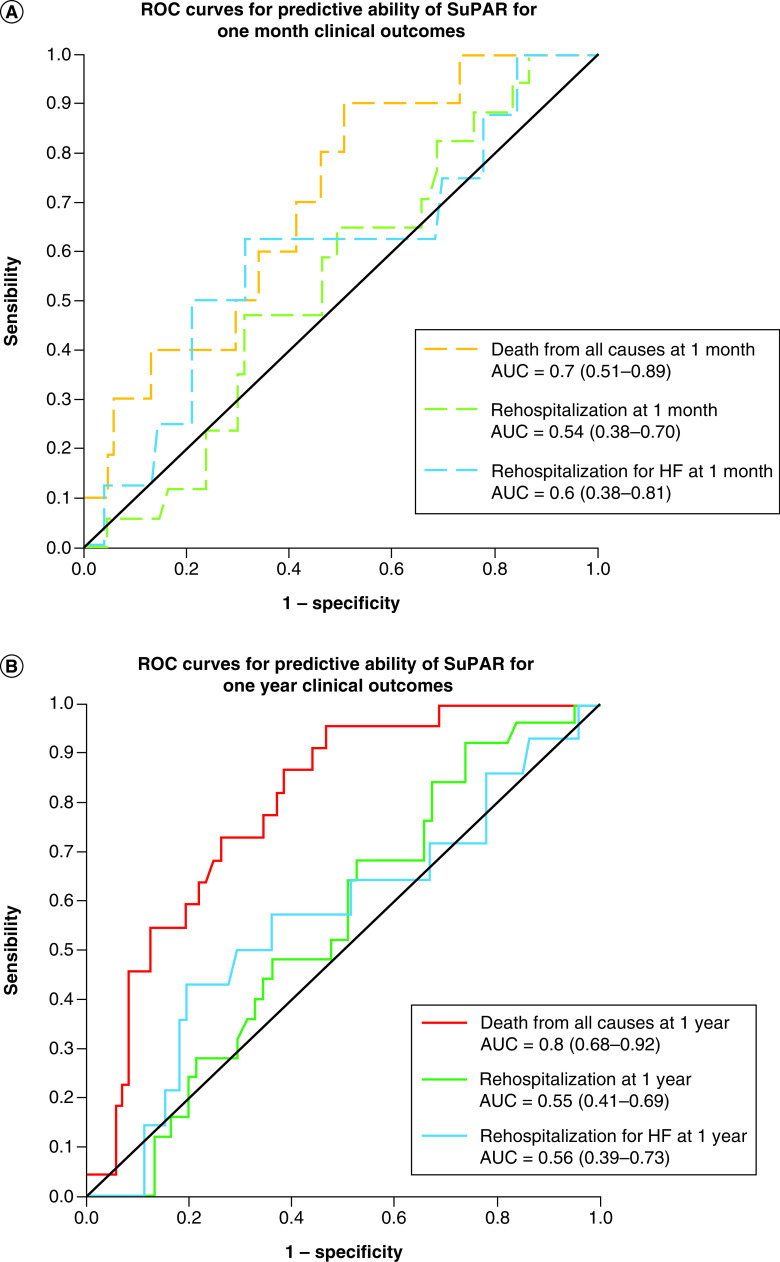
Receiver operating characteristic curves for soluble urokinase-type plasminogen activator receptor prediction of clinical outcomes at 1 month (A) and 1 year (B). A 1 month suPAR predicted the risk of death, hospitalization and hospitalization for heart failure with an estimated AUC of 0.7 (0.51–0.89), 0.54 (0.38–0.70) and 0.7 (0.38–0.81), respectively. A 1 year suPAR predicted the risk of death, hospitalization and hospitalization for heart failure with an estimated AUC of 0.8 (0.68–0.92), 0.55 (0.41–0.69) and 0.56 (0.39–0.73), respectively. AUC: Area under the curve; suPAR: Soluble urokinase-type plasminogen activator receptor.

ROC curves for high suPAR level to predict death of readmission are shown in Figure 2.

### Multivariate analysis for suPAR adjusted with age, LVEF & gender

The multivariate analysis was adjusted for age, gender, and LVEF (Table 2). suPAR was positively associated with the risk of death at one month (p < 0.01) and at one year (p < 0.01), independently of the other variables in the model. Each increase of one suPAR ng/ml was associated with an odds ratio (OR) of 1.28 for death at 1 month (95% CI: 1.07–1.52; p < 0.01) and an OR of 1.31 for death at one year (95% CI: 1.13–1.51). Age was also positively associated with death at one month (p = 0.05) and 1 year (p < 0.01) and LVEF was negatively associated with death at 1 month (p = 0.02) but not at 1 year (p = 0.17). Female gender was significantly associated with death at one year (p = 0.04). suPAR was not associated with the risk of all-cause readmission at one month (p = 0.43) and one year (p = 0.78).

**Table 2.** Multivariate analyses adjusted for clinical factors.

	Adjusted OR for 1 month death		Adjusted OR for 1 month all-cause readmission		Adjusted OR for 1 month heart failure readmission		Adjusted OR for 1 year death		Adjusted OR for 1 year all-cause readmission		Adjusted OR for 1 year heart failure readmission	
	OR (95% CI)	p-value	OR (95% CI)	p-value	OR (95% CI)	p-value	OR (95% CI)	p-value	OR (95% CI)	p-value	OR (95% CI)	p-value
sUPAR	1.28 (1.07–1.52)	0.006	0.94 (0.82–1.09)	0.428	0.60 (0.13–2.75)	0.513	1.31 (1.13–1.51)	<0.001	1.02 (0.91–1.14)	0.782	1.03 (0.9–1.18)	0.656
Age	1.09 (1.00–1.18)	0.047	0.98 (0.94–1.02)	0.233	1 (0.95–1.05)	0.946	1.09 (1.02–1.16)	0.008	0.97 (0.93–1.00)	0.063	0.98 (0.94–1.02)	0.374
Sex (female)	0.25 (0.03–1.98)	0.190	1.08 (0.31–3.71)	0.908	0.73 (0.14–3.93)	0.718	0.2 (0.04–0.90)	0.036	0.9 (0.31–2.61)	0.851	1 (0.29–3.51)	0.996
LVEF	0.93 (0.88–0.99)	0.016	0.99 (0.95–1.03)	0.615	1.01 (0.95–1.07)	0.763	0.97 (0.93–1.01)	0.166	1 (0.97–1.04)	0.958	1.01 (0.97–1.06)	0.640

Logistic model adjusted for suPAR, age, sex and LVEF. Odds ratios are given for 1 unit increase unless indicated by odds ratio for variable higher than median vs above.

LVEF: Left ventricular ejection fraction; OR: Odds ratio; sUPAR: Soluble urokinase-type plasminogen activator receptor.

### Multivariate analysis for suPAR adjustment with other biomarkers to predict the risk of death at 1 year

The multivariate analysis was then adjusted for NTproBNP, CRP, sST2. suPAR was positively associated with the risk of death at 1 year (p = 0.004), independently of the other variables in the model (Table 3). Each increase of one suPAR ng/ml was associated with an OR of 1.20 for death at one year (95% CI: 1.06–1.35; p = 0.004).

**Table 3.** Multivariate analyses adjusted for biological factors.

	Adjusted OR for 1 month death		Adjusted OR for 1 month all-cause readmission		Adjusted OR for 1 month heart failure readmission		Adjusted OR for 1 year death		Adjusted OR for 1 year all-cause readmission		Adjusted OR for 1 year heart failure readmission	
	OR (95% CI)	p-value	OR (95% CI)	p-value	OR (95% CI)	p-value	OR (95% CI)	p-value	OR (95% CI)	p-value	OR (95% CI)	p-value
sUPAR	1.88 (0.95–3.69)	0.069	0.69 (0.33–1.43)	0.313	0.61 (0.12–3.28)	0.567	2.34 (1.31–4.17)	0.004	1.14 (0.66–1.96)	0.650	1.17 (0.60–2.28)	0.645
s–ST2	1.57 (0.67–3.67)	0.303	1.86 (0.55–6.36)	0.320	0.53 (0.20–1.40)	0.199	1.52 (0.86–2.71)	0.151	0.70 (0.40–1.22)	0.210	0.74 (0.39–1.41)	0.361
Nt–proBNP	0.99 (0.58–1.69)	0.966	0.86 (0.44–1.68)	0.652	1.12 (0.59–2.16)	0.727	1.03 (0.65–1.63)	0.906	0.84 (0.47–1.50)	0.554	0.90 (0.47–1.73)	0.748
CRP	2.04 (1–4.14)	0.048	1.03 (0.48–2.19)	0.944	3.02 (0.56–16.5)	0.201	1.35 (0.80–2.29)	0.262	1.31 (0.66–2.57)	0.441	1.69 (0.48–5.95)	0.416

Logistic model adjusted for suPAR, s-ST2, Nt-proBNP and CRP. Odds ratios are given for 1 unit increase unless indicated by odds ratio for variable higher than median vs above.

CRP: C-reactive protein; Nt-proBNP: N terminal pro brain natriuretic peptide; OR: Odds ratio; sUPAR: Soluble urokinase-type plasminogen activator receptor.

## Discussion

The main findings of our study are:

- suPAR levels were independently associated with mortality at 1 month and 1 year for patients with acute HF, but without significant prediction of the hospitalization risk;
- Multivariate Cox analysis highlighted that suPAR was a stronger predictor for mortality than other biomarkers (including sST2, NT-proBNP or CRP) or clinical characteristics.

In the past decade, several prognostic biomarkers have been tested to evaluate HF patients prognosis in the setting of chronic state or decompensated HF, because these biomarkers could reflect distinct pathophysiological pathways including myocardial fibrosis, hypervolemia, myocardial stretch, inflammation or remodeling. CRP is considered classically as a reliable biomarker for systemic inflammation. CRP is produced mainly by hepatocytes but also by cardiovascular tissues upon infection or tissue injury [14,15]. Numerous studies have confirmed that patients with HF present both local and systemic inflammation, and reported that elevated serum levels of CRP is clearly related to cardiovascular events and to mortality independently of natriuretic peptides [16–18]. Recently, sST2 has rapidly emerged because of its pluripotent role in inflammation, mechanical strain, remodeling and fibrosis [12]. sST2 appears promising in prognostic prediction of mortality in patients with chronic HF. In previous study [6] we have confirmed that ST2 alone is an important risk factor for 42-month all-cause or cardiovascular mortality in chronic HF patients. However, the STADE-HF trial failed to demonstrate a reduction in cardiac rehospitalization after an admission for acute HF [12].

In the field of biomarkers of global prognosis, our team has demonstrated that sST2 and GDF-15 can significantly improve the prognosis evaluation of HF patients [19]. This biomarkers could help us to guide therapy and non-pharmacologic adaptations (recurrent rehabilitations, integrative programs, etc.) [20]. Others could help to tailor more and more individualized approaches. In line, several studies had already demonstrated that high suPAR level was linked to the risk of both cardiovascular events and all-cause mortality in the global population [21]. Inflammatory processes are strongly associated with acute and chronic heart failure. Pro-inflammatory cytokines are promoting cardiac hypertrophy, fibrosis and apoptosis, leading to ventricular dysfunction and heart failure [22]. When inflammatory cells are activated by cytokines, the expression of uPAR is upregulated, released into the extra cellular matrix in a soluble form, thus suPAR level increases in the blood. suPAR reflects then an immune activation and is increased in various pathologies settings as cancer, diabetes, infection as well as cardiovascular diseases [23]. However, this biomarkers could provide different information from CRP. Indeed, suPAR has been reported to provide prognostic information in patient with HF [24], but was also able to predict accurately all-cause mortality and recurrent myocardial infarction (MI) after primary myocardial ischemic event, whereas CRP failed in this indication [22]. Similarly, suPAR was independently associated with subsequent new-onset AF in a population of recently hospitalized patients [25]. The concept of exploring different biomarkers involved in inflammation by different pathways could help to understand the pathophysiology of the disease but also in the therapeutic decision-making. Here, we found that suPAR was significantly associated with long term mortality risk on multivariate analysis, but was not associated with the risk of hospitalization, while CRP, NTproBNP and ssT2 proved to be effective to this purpose. suPAR was a better prognostic tool over sST2 and was predictive of adverse outcome after adjustment on clinical confounders, over other biomarkers.

In other words, suPAR could provide additional information to natriuretic peptide, CRP and sST2, all these biomarkers could be proposed as parts of a kind of biological Swiss knife, in order to adequately tailor individualized management of patients with HF [26]. Indeed, suPAR could ultimately provide information about patient prognosis over the cardiological status, as suggested by the fact that it failed to predict the risk of cardiac re admission.

## Conclusion

High suPAR levels during hospitalization for acute HF were highly predictive for the risk of mortality, but not the risk of readmission. These observations emphasize the importance of suPAR measurement on initial presentation in patients with suspected HF, to identify high risk patients who will need short term follow-up and intensive treatment titration.

## Future perspective

Here is demonstrated that high suPAR levels during hospitalization for acute HF were highly predictive for the risk of mortality, but not readmission. Multi-inflammatory biomarkers is interesting because it provides complementary information for prognosis stratification. The concept of exploring different biomarkers involved in inflammation by different pathways could help in understanding the pathophysiology of the disease but also in the therapeutic decision-making. The pathophysiology of HF is multifactorial and combines local and systemic inflammation. Biomarkers specifically targeting one metabolic pathway are needed and could improve the understanding of this heterogeneous pathology. However, suPAR seems to be some kind of global biomarker. Our team is exploring this biomarker in severe acute heart failure and cardiogenic shock as well. All these biomarkers could be proposed as parts of a kind of biological ‘Swiss knife’, in order to adequately tailor individualized management of patients with HF.

**Summary points**

- Heart failure (HF) is a leading cause of hospitalization, morbidity and mortality, and represents a tremendous burden to healthcare systems.
- Hospital (re)admissions remain frequent and the main determinant of costs and identifying patients at risk of readmission or poor outcome is challenging.
- Urokinase-type plasminogen activator receptor (UPAR) is a glycoprotein involved in tissue reorganization events and regulation of plasminogen. Under inflammatory stimulation the protein detaches from cell surface and transform into its soluble form (soluble urokinase-type plasminogen activator receptor [suPAR]), increasing its serum level.
- We used the cohort of patients from the STADE-HF (Soluble Suppression of Tumorigenesis-2 [sST2] as a Help for Management of Diagnosis, Evaluation and Management of HF) trial. Every patient was hospitalized for acute heart failure.
- Ninety-five patients were included in the statistical analysis.
- At 1 month, the suPAR level of expression was significantly higher in the group of patients who died (7.90 ± 4.35 ng/ml vs 11.94 ± 6.86 ng/ml; p < 0.05), but there was no significant difference according to the readmission on the first month (8.09 ± 4.58 ng/ml vs 6.97 ± 3.18; p = 0.61); similarly no difference according readmission for heart failure within the first month (8.01 ± 4.41 ng/ml vs 6.53 ± 3.68; p = 0.37, respectively).
- The multivariate analysis was then adjusted for NTproBNP, CRP, sST2. suPAR was positively associated with the risk of death at one year (p = 0.004), independently of the other variables in the model. Each increase of one suPAR ng/mL was associated with an odds ratio of 1.20 for death at one year (95% CI: 1.06–1.35; p = 0.004).
- In the past decade, several prognostic biomarkers have been tested to evaluate HF patients prognosis in the setting of chronic state or decompensated HF, because these biomarkers could reflect distinct pathophysiological pathways including myocardial fibrosis, hypervolemia, myocardial stretch, inflammation or remodeling.
- sST2 and GDF-15 can significantly improve the prognosis evaluation of HF patients.
- High suPAR level was linked to the risk of both cardiovascular events and all-cause mortality in the global population
- The concept of exploring different biomarkers involved in inflammation by different pathways could help to understand the pathophysiology of the disease but also in the therapeutic decision-making.
- All these biomarkers could be proposed as parts of a kind of biological Swiss knife, in order to adequately tailor individualized management of patients with HF.

## References

[1] Ponikowski P, Voors AA, Anker SD 2016 ESC Guidelines for the diagnosis and treatment of acute and chronic heart failure: The Task Force for the diagnosis and treatment of acute and chronic heart failure of the European Society of Cardiology (ESC). Developed with the special contribution of the Heart Failure Association (HFA) of the ESC. Eur. J. Heart Fail. 18(8), 891–975 (2016). 27207191 10.1002/ejhf.592

[2] Pocock SJ, Ariti CA, McMurray JJV Predicting survival in heart failure: a risk score based on 39 372 patients from 30 studies. Eur. Heart J. 34(19), 1404–1413 (2013). 23095984 10.1093/eurheartj/ehs337

[3] Wang TJ, Wollert KC, Larson MG Prognostic utility of novel biomarkers of cardiovascular stress: the Framingham Heart Study. Circulation 126(13), 1596–1604 (2012). 22907935 10.1161/CIRCULATIONAHA.112.129437 PMC3656719

[4] AbouEzzeddine OF, McKie PM, Dunlay SM Suppression of tumorigenicity 2 in heart failure with preserved ejection fraction. J. Am. Heart Assoc. 6(2), 2017). 10.1161/JAHA.116.004382 PMC5523750 28214792

[5] Aimo A, Vergaro G, Passino C Prognostic value of soluble suppression of tumorigenicity-2 in chronic heart failure: a meta-analysis. JACC Heart Fail. 5(4), 280–286 (2017). 27816512 10.1016/j.jchf.2016.09.010

[6] Dupuy AM, Curinier C, Kuster N Multi-marker strategy in heart failure: combination of ST2 and CRP predicts poor outcome. PLoS ONE 11(6), e0157159 (2016). 27311068 10.1371/journal.pone.0157159 PMC4911159

[7] Briasoulis A, Androulakis E, Christophides T, Tousoulis D. The role of inflammation and cell death in the pathogenesis, progression and treatment of heart failure. Heart Fail. Rev. 21(2), 169–176 (2016). 26872673 10.1007/s10741-016-9533-z

[8] Dupuy AM, Kuster N, Curinier C Exploring collagen remodeling and regulation as prognosis biomarkers in stable heart failure. Clin. Chim. Acta Int. J. Clin. Chem. 490, 167–171 (2019). 10.1016/j.cca.2018.08.042 30179616

[9] Kuster N, Huet F, Dupuy A Multimarker approach including CRP, sST2 and GDF-15 for prognostic stratification in stable heart failure. ESC Heart Fail. 7(5), 2230–2239 (2020). 32649062 10.1002/ehf2.12680 PMC7524044

[10] Winnicki W, Sunder-Plassmann G, Sengölge G Diagnostic and prognostic value of soluble urokinase-type plasminogen activator receptor (suPAR) in focal segmental glomerulosclerosis and impact of detection method. Sci. Rep. 9(1), 1–9 (2019). 31551522 10.1038/s41598-019-50405-8 PMC6760112

[11] Winnicki W, Sunder-Plassmann G, Sengölge G Diagnostic and prognostic value of soluble urokinase-type plasminogen activator receptor (suPAR) in focal segmental glomerulosclerosis and impact of detection method. Sci Rep 9, 13783 (2019). 31551522 10.1038/s41598-019-50405-8 PMC6760112

[12] Huet F, Nicoleau J, Dupuy A-M STADE-HF (sST2 As a help for management of HF): a pilot study. ESC Heart Fail. 7(2), 774–778 (2020). 32168423 10.1002/ehf2.12663 PMC7160465

[13] Curinier C, Solecki K, Dupuy A-M Evaluation of the sST2-guided optimization of medical treatments of patients admitted for heart failure, to prevent readmission: study protocol for a randomized controlled trial. Contemp. Clin. Trials 66, 45–50 (2018). 29414143 10.1016/j.cct.2018.01.007

[14] Libby P. Inflammation in atherosclerosis. Nature 420(6917), 868–874 (2002). 12490960 10.1038/nature01323

[15] Libby P, Ridker PM, Maseri A. Inflammation and atherosclerosis. Circulation 105(9), 1135–1143 (2002). 11877368 10.1161/hc0902.104353

[16] Heart Protection Study Collaborative Group, Jonathan E, Derrick B C-reactive protein concentration and the vascular benefits of statin therapy: an analysis of 20,536 patients in the Heart Protection Study. Lancet Lond. Engl. 377(9764), 469–476 (2011). 10.1016/S0140-6736(10)62174-5 PMC3042687 21277016

[17] Van Tassell BW, Abouzaki NA, Oddi Erdle C Interleukin-1 blockade in acute decompensated heart failure: a randomized, double-blinded, placebo-controlled pilot study. J. Cardiovasc. Pharmacol. 67(6), 544–551 (2016). 26906034 10.1097/FJC.0000000000000378 PMC5749643

[18] Ridker PM, Everett BM, Thuren T Antiinflammatory therapy with canakinumab for atherosclerotic disease. N. Engl. J. Med. 377(12), 1119–1131 (2017). 28845751 10.1056/NEJMoa1707914

[19] Dupuy AM, Kuster N, Bargnoux AS Long term pronostic value of suPAR in chronic heart failure: reclassification of patients with low MAGGIC score. Clin. Chem. Lab. Med. (2021). 10.1515/cclm-2020-0903 33544524

[20] Mirna M, Lichtenauer M, Wernly B Novel cardiovascular biomarkers in patients with cardiovascular diseases undergoing intensive physical exercise. Panminerva Med. 62(3), 135–142 (2020). 32309918 10.23736/S0031-0808.20.03838-0

[21] Eugen-Olsen J, Andersen O, Linneberg A Circulating soluble urokinase plasminogen activator receptor predicts cancer, cardiovascular disease, diabetes and mortality in the general population. J. Intern. Med. 268(3), 296–308 (2010). 20561148 10.1111/j.1365-2796.2010.02252.x

[22] Lyngbæk S, Marott JL, Møller DV Usefulness of soluble urokinase plasminogen activator receptor to predict repeat myocardial infarction and mortality in patients with ST-segment elevation myocardial infarction undergoing primary percutaneous intervention. Am. J. Cardiol. 110(12), 1756–1763 (2012). 22981263 10.1016/j.amjcard.2012.08.008

[23] Hamie L, Daoud G, Nemer G SuPAR, an emerging biomarker in kidney and inflammatory diseases. Postgrad. Med. J. 94(1115), 517–524 (2018). 30177549 10.1136/postgradmedj-2018-135839

[24] Koller L, Stojkovic S, Richter B Soluble urokinase-type plasminogen activator receptor improves risk prediction in patients with chronic heart failure. JACC Heart Fail. 5(4), 268–277 (2017). 28359415 10.1016/j.jchf.2016.12.008

[25] Oscar W, Line Jee Hartmann R, Ove A, Eric B, Jesper Eugen O, Jens F. Soluble urokinase plasminogen activator receptor (suPAR) as a predictor of incident atrial fibrillation. J. Atr. Fibrillation 10(6), 1801–1801 (2018). 29988279 10.4022/jafib.1801 PMC6009789

[26] Dalos D, Spinka G, Schneider M New cardiovascular biomarkers in ischemic heart disease-GDF-15, a probable predictor for ejection fraction. J. Clin. Med. 8(7), 924 (2019). 10.3390/jcm8070924 PMC6678676 31252588

